# Redefining bladder cancer treatment: innovations in overcoming drug resistance and immune evasion

**DOI:** 10.3389/fimmu.2025.1537808

**Published:** 2025-01-22

**Authors:** Peng Huang, Jie Wang, Zongze Yu, Jiaan Lu, Zhou Sun, Zhigui Chen

**Affiliations:** ^1^ Department of Urology, The Second People's Hospital of Meishan City, Meishan, Sichuan, China; ^2^ Clinical Medical College, Southwest Medical University, Luzhou, China; ^3^ Department of Urology, China-Japan Union Hospital of Jilin University, Changchun, Jilin, China

**Keywords:** bladder cancer, drug resistance, tumor microenvironment, precision therapy, combined therapy

## Abstract

Bladder cancer is one of the most common malignancies of the urinary system and has always presented great challenges in treatment due to its intricate biological features and high recurrence rates. Although great developments were achieved in immunotherapy and targeted therapies within the last decade, therapeutic outcomes for a great number of patients remain unsatisfactory, particularly as to long-term efficacy. Review discusses the molecular mechanisms developed during the process of bladder cancer progression: genetic and epigenetic alterations, dynamics of the tumor microenvironment (TME), and dysregulation and abnormal activation of various signaling pathways—all contributing to therapeutic resistance. It is genetic mutation, especially in both low- and high-grade tumors, that, alongside epigenetic modifications, plays a considerable role in tumor aggressiveness and drug resistance. TME, comprising cancer-associated fibroblasts (CAFs), immunosuppressive cells, and different components of the extracellular matrix (ECM), orchestrates a setting that fosters tumor growth and immune evasion and confers resistance on any therapeutic regime that might be used. The review also provides an overview of PI3K/AKT and MAPK signaling pathways in the progression of bladder cancer and the development of targeted therapies against them. Further, it discusses the challenges and mechanisms of resistance to immunotherapy, including those involving immune checkpoint inhibitors. Other promising approaches include the development of new therapeutic strategies that target not only the signaling pathways but also immune checkpoints in combination therapies. This review aims to contribute to the elaboration of more effective and personalized treatment strategies by fully understanding the underlying mechanisms involved in bladder cancer.

## 1 Introduction

Bladder cancer is one of the most common malignant tumors in the urinary system. Such complex biological features and a high recurrence rate have posed great challenges to clinical treatment. More than 70% of bladder cancers are nonmuscle-invasive bladder cancers, and the recurrence rate within five years can reach as high as 50% (1). In the last decade, some advances have been made in immunotherapies and targeted therapies, but the therapeutic responses of most patients are still far from optimal, especially regarding the maintenance of long-term efficacy of the treatment (2). This further underlines the need for in-depth studies on molecular mechanisms and drug resistance in bladder cancer. This review will discuss tumor progression driven by genetics, the microenvironment, and signaling pathways in an attempt to explain the causes for resistance to immunotherapies, while presenting novel strategies to combat drug resistance. These in turn will contribute to the elaboration of more tailored treatment protocols offering improved prognosis for patients in the future.

## 2 Molecular mechanisms of bladder cancer progression

### 2.1 Genetic and epigenetic variations

The cascade leading to bladder cancer progression is rooted in a labyrinth of complex genetic and epigenetic transformations. These cumulative alterations not only amplify the tumor’s aggressiveness but also fortify its resistance to therapeutic interventions.

Diverse characteristics, such as genetic mutations in bladder cancer, vary significantly across different grades. Broadly speaking, low-grade bladder cancers are often associated with several driver mutations. In stark contrast, high-grade bladder cancers frequently exhibit intricate gene alterations and more aggressive mutation profiles (3).This is understandable; tumors with higher malignancy often have a higher mutation burden or gene mutations at more critical sites (4). Based on whether they infiltrate the muscle layer, we have summarized some gene mutations highly associated with low/high-grade bladder cancer (**Table 1**).

**Table 1:** Highly related genetic mutation sites of bladder cancer and their basic interaction pathways and impacts.

Gene	Mutation Details	Pathway Impacted	Function in Cancer	Prognostic Implications	References	Tumor Grade
FGFR3	S249C, Y373C - Commonly drives RAS-MAPK signaling, leading to increased cell proliferation and reduced differentiation. Frequently observed in low-risk NMIBC cases.	RAS-MAPK	Stimulates cell proliferation, lowers differentiation; favorable prognosis if isolated.	Associated with improved outcomes in NMIBC; may not progress to invasive stages.	(66)	Low-grade Bladder Cancer (NMIBC)
PIK3CA	E542K, E545K, H1047R - Activates PI3K-AKT pathway, enhancing cellular survival and metabolic activity. Often co-mutated with FGFR3 in low-grade cases to potentiate proliferation.	PI3K-AKT	Enhances cell survival and metabolism, amplifies oncogenic signaling in cooperation with FGFR3.	Linked with proliferation but low risk if in combination with FGFR3 in NMIBC.	(67)	
STAG2	LOF mutations - Affect DNA repair and cohesion complex stability, essential for early cell cycle regulation and stability. Mutations correlate with early-stage progression but low aggressiveness.	Genomic Stability	Maintains genomic integrity; mutations often promote early tumor growth but without invasion.	Early tumor progression marker but typically indicates non-invasive behavior.	(68, 69)	
TP53	R248, R175, G245 - Mutations in TP53 inhibit cell cycle arrest and apoptosis, leading to genomic instability and aggressive phenotypes in high-grade MIBC.	Cell Cycle	Key tumor suppressor; loss leads to high-grade, invasive, and chemotherapy-resistant tumors.	Strongly linked with poor outcomes, invasiveness, and increased mutation burden.	(66)	High-grade Bladder Cancer (MIBC)
RB1	R661, R698 - Loss of RB1 results in deregulation of cell cycle control, promoting unchecked cellular proliferation. Often co-mutated with TP53, leading to high-grade and recurrence risk.	Cell Cycle	Critical in cell cycle control; loss promotes malignancy and correlates with recurrence.	Predictive of poor prognosis and recurrence in high-grade MIBC.	(70, 71)	
ERBB2	S310F, V777L - Mutations in ERBB2 (HER2) enhance signaling for cell proliferation and invasiveness, often contributing to therapy resistance.	HER2/EGFR	Proto-oncogene; mutations lead to invasive tumor behavior and resistance to certain treatments.	Correlated with aggressive behavior, invasive phenotype, and therapeutic resistance.	(72)	
ARID1A	Q456, R1276 - Loss-of-function in ARID1A affects chromatin structure and gene regulation, linked with high-grade undifferentiated tumors and poor prognosis.	Chromatin Remodeling	Chromatin regulator; mutation leads to dysregulation in gene expression, especially in high-grade tumors.	Indicates high malignancy, undifferentiated growth, and unfavorable outcomes.	(73)	
KMT2D	R2900, Q3432 - Disrupts histone methylation patterns, promoting oncogenic transcriptional programs. Associated with aggressive, treatment-resistant phenotypes.	Histone Methylation	Regulates transcription; loss promotes aggressive tumor characteristics and therapy resistance.	Associated with poor response to therapy and increased mortality.	(74)	

Epigenetic changes—including DNA methylation, histone modifications, abnormal expression of non-coding RNAs, and aberrant expression of DNA demethylases and histone-modifying enzymes—further increase tumor heterogeneity, making therapeutic targeting more difficult (5–7).

### 2.2 Tumor microenvironment

It is in the tumor microenvironment, in the context of bladder cancer, that such complexities arise to unmistakably affect, at a very basic level, the features of cancer growth and progression, as well as resistance to therapy. The TME in itself is a highly heterogeneous cell population and the Extracellular Matrix (ECM) matrix, including Cancer-Associated Fibroblasts (CAFs), immunosuppressive cells, and different ECM components. It is anything but a passive background provided by the tumor cells. Instead, it creates a dynamic and interactive landscape where these components contribute to reinforcing not only the structural tumor but also in altering the behavior itself. Intricate interplay has an effect on the invasiveness of cancer cells, makes them capable of immune evasion, and determines responsiveness to various therapeutic modalities.

#### 2.2.1 CAFs

CAFs are key stromal cells within the TME of bladder cancer, promoting tumor growth, invasion, and metastasis by secreting various growth factors and cytokines. Research indicates that CAFs play a dual role in tumor progression by secreting transforming growth factor-beta (TGF-β) and matrix metalloproteinases (MMPs). TGF-β is a crucial cytokine that can induce epithelial-mesenchymal transition (EMT), enabling bladder cancer cells to acquire enhanced invasiveness and migratory capacity. MMPs degrade extracellular matrix components, creating pathways for tumor cell metastasis and assisting cancer cells in breaching the basement membrane, further promoting cancer spread (8).

Additionally, CAFs release chemokines that attract other immunosuppressive cells—such as regulatory T cells and M2-type macrophages—to the tumor microenvironment, further weakening the body’s anti-tumor immune response. These mechanisms not only make CAFs significant pro-tumor cells in the TME but also underscore their critical role in the poor prognosis of patients with malignant bladder cancer (9).

#### 2.2.2 Immunosuppressive cells

The TME of bladder cancer is likewise highly loaded with different immunosuppressive cells, including regulatory T cells or Tregs and M2-type macrophages. Tregs suppress the activity of effector T cells through the secretion of immunosuppressive cytokines such as IL-10 and TGF-β, which lessens the anti-tumoral response. M2-type macrophages participate in the secretion of several pro-tumoral factors, including vascular endothelial growth factor (VEGF), a factor that promotes the proliferation and survival of tumor cells through angiogenesis and increased supply of nutrients (10).

These immunosuppressive cells synergistically constitute the immune suppressive microenvironment of bladder cancer, which enables cancer cells to effectively evade host immunity and enhances the difficulties in treatment. This immune evasion phenomenon also partly reduces the efficacy of immune checkpoint inhibitors, leading to poor response rates of bladder cancer to immunotherapy (8). Conversely, blocking the inhibitory mechanisms that target these immunosuppressive cells or combining immunosuppressive cell inhibitors with immune checkpoint inhibitors represents a highly promising therapeutic strategy (11).

#### 2.2.3 ECM

In the TME of bladder cancer, the extracellular matrix plays an active role rather than a passive structural role in the interrelationship between cancer cells and the TME. Often in patients with bladder cancer, for example, the structure of the ECM is remodeled through, among other methods, degradation mediated by enzymes such as MMPs. This reconstitution enables the ECM to facilitate routes of migration and dissemination for the tumor cells. Also, such a remodeled ECM could interact with tumor cell surface receptors to trigger the pro-proliferative signaling pathways, such as PI3K-AKT, which eventually supports cancer cell growth and survival (9).

Alterations in ECM structure also significantly affect the effectiveness of bladder cancer treatment. ECM remodeling increases tissue density, creating a physical barrier that may hinder the penetration of chemotherapy drugs and immune cells, thus reducing the efficacy of drugs and immunotherapies. Consequently, disrupting the ECM barrier has become an important research focus in bladder cancer treatment strategies (10). For example, studies have demonstrated that exercise can inhibit tumor ECM, thereby activating the antitumor effects of immune cells (12).

### 2.3 Abnormal activation of signaling pathways

In the development and malignant progression of bladder cancer, the PI3K/AKT and MAPK signaling pathways play crucial roles. The abnormal activation of these pathways drives tumor cell proliferation, invasion, and survival, providing a molecular basis for bladder cancer. Targeted blockade of these signaling pathways, especially in patients with specific pathway mutations, is considered a potential therapeutic strategy.

#### 2.3.1 PI3K/AKT signaling pathway

The PI3K/AKT pathway has been demonstrated to drive cell proliferation and anti-apoptotic properties in various cancers. Studies have shown that in bladder cancer, activation of this pathway is mainly associated with the loss of PTEN (phosphatase and tensin homolog) or PIK3CA gene mutations (**Table 1**), and is more common in high-grade bladder cancer. PTEN is a major negative regulator of the PI3K/AKT pathway; when its function is lost, AKT becomes continuously activated, ultimately promoting cancer cell proliferation and survival. Activation of AKT not only enhances cells’ anti-apoptotic ability but also increases cancer cells’ resistance to chemotherapy drugs, making them more refractory during treatment (13).

#### 2.3.2 MAPK signaling pathway

The MAPK signaling pathway is a primary regulatory pathway for bladder cancer cell proliferation and angiogenesis. This pathway includes multiple cascade kinases, where RAS activates downstream key molecules such as RAF, MEK, and ERK. By regulating the expression of cell cycle proteins, this pathway promotes cell proliferation. Simultaneously, by inducing the production of VEGF, it enhances the tumor’s angiogenic capability, allowing the tumor to obtain more nutritional support (14). In bladder cancer, the MAPK pathway is highly associated with FGFR3 mutations (**Table 1**), commonly observed in low-grade bladder cancer but also present in some patients with high-grade invasive bladder cancer, thereby enhancing the tumor’s invasiveness and metastatic potential (15).

Given the central role of the PI3K/AKT and MAPK signaling pathways in bladder cancer progression, therapeutic strategies targeting these pathways have been extensively researched. PI3K/AKT inhibitors (such as BEZ235 and BKM120) (16), and inhibitors targeting the MAPK pathway (such as MEK and ERK inhibitors) (17), have shown potential in inhibiting bladder cancer cell growth in preclinical models. By blocking downstream signal transduction of PI3K/AKT, these inhibitors reduce tumor cell proliferation rates and induce apoptosis. They can significantly decrease the invasiveness of bladder cancer cells, making them especially suitable for patients with FGFR3 mutations or abnormal activation of the MAPK pathway.

## 3 Immunotherapy of bladder cancer

Immunotherapy, especially checkpoint inhibitors targeting PD-1 and PD-L1, has become a significant advancement in the treatment of bladder cancer. However, there is considerable variation in patient responses to treatment, and the frequent occurrence of resistance poses a major challenge to therapeutic efficacy.

### 3.1 Traditional immunotherapy approaches

Immune checkpoint inhibitors have demonstrated efficacy in bladder cancer patients unresponsive to chemotherapy. For example, pembrolizumab and atezolizumab have been approved by the FDA (18), but only a small portion of patients achieve durable therapeutic responses, indicating the need for predictive biomarkers to optimize patient selection (19).

### 3.2 Mechanisms of resistance

Resistance to immunotherapy arises from multiple intrinsic and extrinsic tumor factors. Tumor cells can downregulate PD-L1 expression or employ other immune checkpoints (such as CTLA-4) to evade immune recognition (20). Additionally, the presence of myeloid-derived suppressor cells (MDSCs) and tumor-associated macrophages (TAMs) within the tumor microenvironment creates an immunosuppressive setting, which significantly dampens T-cell activity and contributes to therapeutic resistance. Specifically, in the tumor microenvironment, MDSCs and M2-type TAMs contribute through:

1. Secreting various immunosuppressive cytokines to inhibit T cells (21).
2. Expressing immune checkpoint molecules (like PD-L1) or promoting upregulation of checkpoint receptors (such as PD-1 and CTLA-4) on T cells, leading to T-cell “exhaustion” (21).
3. Depriving T cells of nutrients and energy (L-arginine and L-tryptophan) and producing excessive reactive oxygen species (ROS) that interfere with T-cell metabolism (22).

This process allows tumor cells to continue proliferating under the “umbrella” of the microenvironment.

### 3.3 Emerging immunotherapeutic strategies

**Figure 1:**
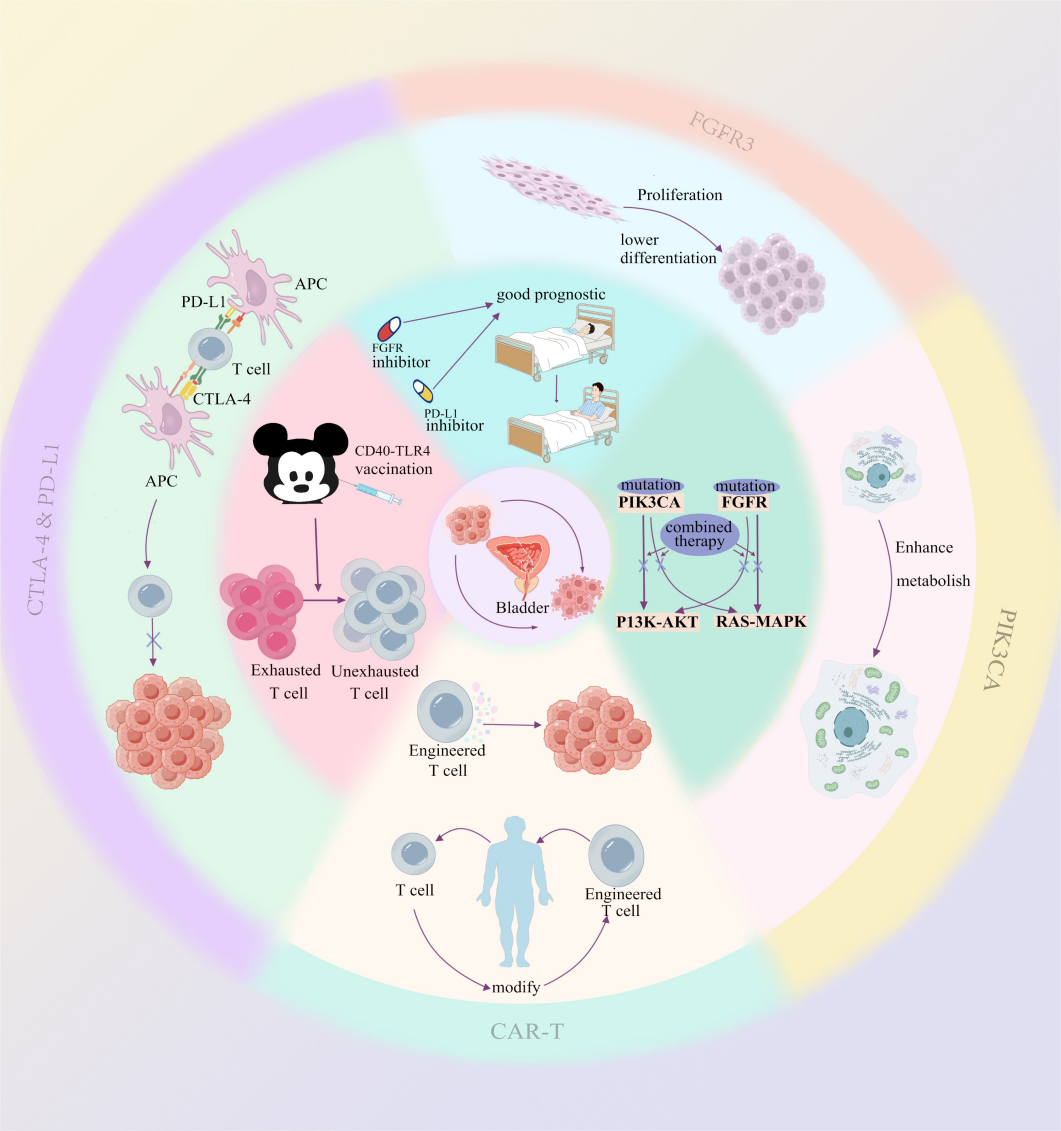
The outermost circle and the second circle from the outside inward illustrate the direct impact of each mechanism on the tumor or tumor microenvironment. The inner circle (the third circle from the outside inward) represents the underlying therapeutic rationale or prognosis associated with multi-mechanism combination therapies.

To overcome resistance, current research is exploring new methods. For instance, CAR-T cell therapy is gradually being adapted for solid tumors. Specifically for bladder cancer, researchers have developed novel CAR-T cells targeting SIA-CIgG, which have shown effective anti-tumor activity (23) (**Figure 1**).

Furthermore, the exploration and development of immune mechanisms are progressively advancing. Survival analyses conducted using database resources have revealed an association between IL-15 expression and favorable cancer prognosis across multiple cancer types (pan-cancer) (24). Additionally, the production of IL-15 is highly correlated with exercise (25), suggesting that interventions in lifestyle habits (such as physical activity) during the early stages of cancer may influence the overall cancer outcomes. In the future, cancer treatment strategies may extend beyond clinical interventions to include the formulation of plans based on patients’ lifestyle behaviors (26, 27).

## 4 Drug sensitivity and targeted therapy

Traditional chemotherapeutic agents like cisplatin and gemcitabine have certain efficacy in the treatment of bladder cancer, but their toxicity and resistance limit long-term outcomes (28, 29). In recent years, targeted drugs, such as erdafitinib, which targets FGFR mutations, have shown positive efficacy in bladder cancer patients carrying FGFR mutations, demonstrating the potential of biomarker-driven therapies (30).

### 4.1 From drug sensitivity to clinical decision-making

Tailoring treatment based on biomarkers to predict patients’ sensitivity to drugs is crucial for personalized medicine.

1. PIK3CA gene mutations and loss of PTEN can activate the PI3K/AKT signaling pathway; the PIK3CA-targeted inhibitor alpelisib has been used in breast cancer (31).

2. FGFR3 gene mutations can activate the RAS-MAPK signaling pathway. Erdafitinib is an FDA-approved FGFR inhibitor that has shown significant efficacy against bladder cancer with FGFR3 mutations or translocations (30).

3. TP53 and RB1 genes can cause cell cycle dysregulation. APR-246 is an investigational p53 reactivator undergoing trials in different types of cancer, showing certain promise (32). In tumors with RB1 mutations, CDK4/6 inhibitors (such as palbociclib) may indirectly control the cell cycle (33).APR-246 and CDK4/6 inhibitors could provide a synergistic effect by targeting both p53 and cell cycle pathways, as demonstrated in preclinical models and ongoing trials (34).

4. CTLA-4 and PD-L1 act on immune checkpoints to induce immunosuppression, suggesting sensitivity to their corresponding inhibitors.

5. High tumor mutational burden (TMB) and microsatellite instability-high (MSI-H) make tumors more immunogenic, indicating sensitivity to immune checkpoint inhibitors (ICIs) (35).

6. The application prospects of ERBB2 (HER2) inhibitors, common in breast cancer treatment strategies, are also receiving much attention in bladder cancer. New multitargeted ERBB2 family inhibitors, such as afatinib and dacomitinib, show potential for treating tumors resistant to traditional HER2-targeted therapies by irreversibly inhibiting multiple ERBB2 receptors. These inhibitors may offer an advantage in tumors expressing multiple ERBB2 receptors, such as bladder cancer (36).

However, even with targeted therapy, the issue of resistance still exists.

### 4.2 Mechanisms of resistance to single-target therapy

#### 4.2.1 Bypass Activation of Signaling/Cell Cycle Pathways

Even when using PIK3CA inhibitors (such as alpelisib) to inhibit PI3K/AKT, tumor cells may continue to proliferate by activating other pathways such as MAPK or JAK/STAT (37). Similarly, using erdafitinib to target FGFR3 mutations, activation of other pathways may allow tumor cells to continue growing under FGFR3 inhibition (38). Both TP53 and RB1 can activate compensatory cell cycle pathways through bypass mechanisms, and the cell cycle network regulated by RB1 is extremely complex. This complexity makes it difficult for single CDK4/6 inhibitors to completely stop cell proliferation, which explains why targeted therapies rarely focus solely on RB1 (39–41). Additionally, in HER2-positive breast cancer cells, tumor cells may escape ERBB2 inhibition through bypass signaling pathways such as PI3K/AKT and MAPK, enhancing the growth and proliferation of tumor cells. Activation of these bypass pathways is a major factor in treatment failure (42).

#### 4.2.2 Adaptive Changes in Signaling Pathways

Mutations in downstream molecules may lead to continuous activation of signaling pathways, thereby reducing the efficacy of PI3K inhibitors and drugs targeting FGFR3 mutations (43, 44).

#### 4.2.3 Resistance to Immune Checkpoint Inhibitors

Even when using CTLA-4 and PD-L1 inhibitors, immunosuppressive cells in the tumor microenvironment (such as MDSCs and Tregs) can hinder T-cell activity by secreting immunosuppressive factors like TGF-β and IL-10, allowing tumors to evade immune attack (45). Studies have also found that tumor cells may escape the immune system by upregulating other immune checkpoints such as TIM-3 and LAG-3, increasing drug resistance (46). Moreover, long-term immunotherapy can lead to T-cell exhaustion, causing them to gradually lose the ability to attack cancer cells, resulting in therapeutic resistance (47).

#### 4.2.4 Immunotherapy Resistance in High TMB and MSI-H Tumors

Although MSI-H tumors with high mutation burdens produce a great number of neoantigens, studies on colorectal cancer have demonstrated that tumors may develop antigen presentation defects due to the loss of β2-microglobulin (B2M), weakening the immune response (48). In some tumors with high TMB, despite the presence of high-affinity neoantigens, their low expression levels lead to ineffective T-cell recognition and response to tumor cells (49). Similarly, as immune pathway targeting points, high TMB and MSI-H can also be undermined by the upregulation of other immune checkpoints like LAG-3 and TIM-3 and by T-cell exhaustion, as mentioned above.

### 4.3 Emerging combination therapies

Under the heavy burden of drug resistance, finding effective treatment methods to overcome resistance has become a top priority.

#### 4.3.1 Combination of signaling pathway inhibitors/combination of cell cycle inhibitors

Many studies have found that regardless of inhibiting cell cycle pathways or signaling pathways, bypass activation remains a very common and important mechanism of resistance. Therefore, combined therapeutic strategies targeting this mechanism may have very broad prospects. This strategy has been proven to be significant not only in bladder cancer but also in head and neck squamous cell carcinoma (50) and clear cell ovarian carcinoma (51). The combined use of PI3K inhibitors and MEK inhibitors can significantly inhibit the growth of bladder cancer cells, overcome the resistance of single agents, and exhibit stronger anti-tumor activity than single drugs (52, 53) (**Figure 1**).

#### 4.3.2 Immune checkpoint inhibitors combined with signaling pathway inhibitors

In addition to “blocking” signaling pathways, combining immune checkpoint inhibitors and signaling pathway inhibitors seems to be a very promising method through multiple mechanisms. Mathematical models have shown that combining FGFR inhibitors with anti-PD-L1 therapy can optimize the treatment effect of FGFR-mutant bladder cancer (54). Some studies have also proposed that this scheme has advantages (55) (**Figure 1**).

#### 4.3.3 Reversing T-cell exhaustion—combined activation of CD40 and TLR4

Studies have shown that by combining CD40 and TLR4 stimulation, it is possible to restore the sensitivity of exhausted T cells in PD-1 inhibitor-resistant tumors. Specifically, when CD40 and TLR4 are simultaneously activated, the resulting immune response is not limited to local tumors but also induces systemic anti-tumor T-cell responses. This “*in situ* vaccine” therapy reactivates immune cells within the tumor, allowing effector T cells to enter the tumor microenvironment and enhance their specificity in attacking tumors (56) (**Figure 1**).

## 5 Discussion

### 5.1 The intricate genetic molecular network of bladder cancer

The resistance of bladder cancer—to traditional treatments, immunotherapy, or targeted therapy—resembles a complex intertwined network, with overlapping mechanisms yet distinct differences. So far, we have preliminarily revealed the roles of genetic variations, tumor microenvironment, and abnormal signaling pathways in the progression of bladder cancer.

At the genetic level, efforts to discover unidentified key genes and mutation sites—starting from signaling networks and cell cycles—will become easier to achieve with the support of sufficient gene expression databases. Identifying more combined targets can facilitate the development of innovative treatment plans.

For epigenetic mutations, directly targeting or treating them is relatively difficult, specifically because:

1. Epigenetic modifications not only act on cancer cells but also affect various cell types in the tumor microenvironment (such as immune cells and stromal cells). Different cell types may respond differently to epigenetic therapy, leading to unpredictable treatment effects (57, 58). Moreover, epigenetic modifications often involve multiple types; they function like a team, relying on each other and acting synergistically, making it difficult to produce significant effects by targeting a single modification (59).
2. Epigenetic modifications exist in both normal and cancer cells, lacking tumor specificity. Epigenetic drugs may affect gene expression in normal cells, leading to unforeseen side effects. For example, DNA methylation inhibitors or histone deacetylase inhibitors (HDAC inhibitors) may cause widespread changes in gene expression, affecting the function of healthy tissues (57, 58).
3. Epigenetic modifications are reversible, which is advantageous to some extent because normal gene expression can be restored through intervention (60). However, this reversibility also allows cancer cells to adjust their modification state through feedback mechanisms, thereby developing drug resistance (61).

In summary, discovering the associations between epigenetic changes and various environmental factors, identifying causes and high-risk factors to promote avoidance, is a more cost-effective choice. For instance, by continuously updating large-sample databases and bioinformatics data with population representation, advanced clinical prognostic models can be developed to optimize prevention and early treatment.

### 5.2 Bladder cancer in the tumor microenvironment

Regardless of the type of tumor, the microenvironment in which it resides is always a primary focus of research in its treatment. The immunosuppressive nature of bladder cancer exacerbates treatment difficulties, primarily due to the presence of infiltrating MDSCs and TAMs within its microenvironment. These cells suppress the anti-tumor activity of T cells, leading to poor efficacy of immune checkpoint inhibitors. Future treatments can focus on key components of the TME, such as inhibiting the activity of CAFs, reducing the immunosuppressive effects of Tregs and M2-type macrophages, or increasing drug permeability by altering the structure of the ECM (62, 63).

Moreover, the advancement of CAR-T therapy from hematological tumors to solid tumors also shows considerable development prospects. The development of novel CAR-T cells targeting SIA-CIgG suggests that we can focus on discovering suitable targets to adapt to more solid tumors in the future (23).

In addition, emerging combination therapies that reverse T-cell exhaustion suggest that the tumor microenvironment not only promotes tumors but, under proper guidance and tool utilization, can also be transformed into a “sharp blade” in our hands to combat tumor progression (56).

As the tumor genetic loci, signaling pathways, and immune mechanisms associated with bladder cancer are gradually being elucidated, a diverse array of therapeutic approaches has emerged. Fundamentally, the further advancement of cancer treatment lies in the integration of diagnostic technologies with therapeutic methods. For example, among 100 bladder cancer patients, there may exist 100 distinct subtypes of bladder tumors. The more precisely these patients are classified and the more effectively personalized targeted therapies are administered(Moreover, classification can be based not only on genetic phenotypes and signaling pathway characteristics but also on demographic baseline differences such as gender and age (64), as well as biological differences including metabolic activity (65).), the better the prognoses for the patients will be. This review comprehensively enumerates various targeted loci, as well as personalized treatment regimens and combination therapies based on tumor phenotypes, within this framework. We propose that the future of cancer therapy will ultimately involve extensive classification databases and corresponding treatment regimen databases, facilitating a transition from generalized treatments to personalized therapies.
